# Prion protein and aging

**DOI:** 10.3389/fcell.2014.00044

**Published:** 2014-08-29

**Authors:** Lisa Gasperini, Giuseppe Legname

**Affiliations:** Laboratory of Prion Biology, Department of Neuroscience, Scuola Internazionale Superiore di Studi AvanzatiTrieste, Italy

**Keywords:** PrP, aging, Alzheimer disease, prion diseases

## Abstract

The cellular prion protein (PrP^C^) has been widely investigated ever since its conformational isoform, the prion (or PrP^Sc^), was identified as the etiological agent of prion disorders. The high homology shared by the PrP^C^-encoding gene among mammals, its high turnover rate and expression in every tissue strongly suggest that PrP^C^ may possess key physiological functions. Therefore, defining PrP^C^ roles, properties and fate in the physiology of mammalian cells would be fundamental to understand its pathological involvement in prion diseases. Since the incidence of these neurodegenerative disorders is enhanced in aging, understanding PrP^C^ functions in this life phase may be of crucial importance. Indeed, a large body of evidence suggests that PrP^C^ plays a neuroprotective and antioxidant role. Moreover, it has been suggested that PrP^C^ is involved in Alzheimer disease, another neurodegenerative pathology that develops predominantly in the aging population. In prion diseases, PrP^C^ function is likely lost upon protein aggregation occurring in the course of the disease. Additionally, the aging process may alter PrP^C^ biochemical properties, thus influencing its propensity to convert into PrP^Sc^. Both phenomena may contribute to the disease development and progression. In Alzheimer disease, PrP^C^ has a controversial role because its presence seems to mediate β-amyloid toxicity, while its down-regulation correlates with neuronal death. The role of PrP^C^ in aging has been investigated from different perspectives, often leading to contrasting results. The putative protein functions in aging have been studied in relation to memory, behavior and myelin maintenance. In aging mice, PrP^C^ changes in subcellular localization and post-translational modifications have been explored in an attempt to relate them to different protein roles and propensity to convert into PrP^Sc^. Here we provide an overview of the most relevant studies attempting to delineate PrP^C^ functions and fate in aging.

## Introduction

The conformational conversion of the cellular form of the prion protein (PrP^C^) into a β-sheet enriched isoform denoted as prion (PrP^Sc^) is central in neurodegenerative pathologies collectively know as prion diseases (Prusiner, 2001).

The PrP^C^ is a sialoglycoprotein that is attached to the outer leaflet of the plasma membrane via a C-terminal glycosylphosphatidylinositol (GPI) anchor (Stahl et al., 1990). In the cell PrP^C^ may be expressed in different glycosylated forms, corresponding to the variable occupancy of the residues Asn180 and Asn196 (Haraguchi et al., 1989). While the C-terminal domain is folded in ordered and distinctive secondary structures, the N-terminal portion is flexible, unstructured and contains a unique octapeptide repeat (OR) region (Zahn et al., 2000). The OR confers PrP^C^ one of its most salient features, i.e., the ability to bind divalent cations, prominently copper and to a lesser extent zinc, nickel, iron, and manganese (Stockel et al., 1998; Jackson et al., 2001; Singh et al., 2010; Arena et al., 2012). Indeed, at physiological pH copper shows the highest binding affinity (Chattopadhyay et al., 2005; Liu et al., 2011). The importance of copper binding for PrP^C^ function is reflected in the high OR structural homology among different species (Hornshaw et al., 1995). Two of the most investigated putative functions for PrP^C^ are neuroprotection and defense against oxidative stress, both relevant in neurodegeneration and cell aging (Vassallo and Herms, 2003; Roucou and LeBlanc, 2005; Aguzzi et al., 2008). In agreement with these findings, PrP^C^ copper binding sites support the reduction of Cu^2+^ to Cu^+^, thus preventing reactive oxygen species (ROS) formation (Liu et al., 2011).

Although contrasting results are likely due to the different experimental protocols employed, PrP^C^ is expressed in almost all body organs and tissues, from embryonic to aging stages (Fournier et al., 1998; Goldmann et al., 1999; Sales et al., 2002). However, the expression levels are differently regulated according to the age and tissue distribution. For instance, while PrP^C^ shows a high expression in the central nervous system (CNS), where it is differently regulated during development and aging, it is present at very low levels in the liver (Horiuchi et al., 1995; Moudjou et al., 2001; Peralta et al., 2012). The broad homology conservation of the PrP^C^-encoding gene among mammalian and avian species (Hornshaw et al., 1995), the expression of the protein in many tissues during the entire lifespan, and its high turnover rate strongly indicate that PrP^C^ possesses key physiological roles. However, the definition of PrP^C^'s univocal function is still under debate.

The PrP^Sc^ isoform can form protein aggregates, known as prion deposits, often present as amyloid structures, which can propagate and possibly cause cell death. Despite continuous steps forward toward the definition of the conformational switch mechanism, the trigger of the posttranslational remodeling event is still obscure, especially in sporadic prion disorders. In the inherited forms of prion diseases, a genetic mutation in the open-reading frame of the gene leads to amino acid substitutions, which in turn destabilizes the protein structure over time, promoting PrP^C^ to PrP^Sc^ conversion. In the infective forms, a preformed PrP^Sc^-aggregate triggers the conversion of endogenous PrP^C^. In the sporadic forms of the disease, several unknown factors may perturb the protein structure, thus favoring PrP^Sc^ formation (Prusiner, 1991, 1994).

If a cause-effect relationship between prion deposits and cell death does not always exist (Lasmezas et al., 1997), a probable consequence of PrP^C^ conversion into PrP^Sc^ is the loss or alteration of its cellular function. Whether this impairment contributes or not to disease development is still under debate. Moreover, PrP^C^ is also involved in Alzheimer disease (AD) (Kellett and Hooper, 2009). Whether in AD PrP^C^ mediates neuroprotection or β-amyloid toxicity is still under investigation.

All these observations, together with the fact that the highest incidence of prion disorders and AD is in the elderly population, have renewed the interest of scientists in investigating of the role of PrP^C^ during aging. The two main topics under study have been: (i) PrP^C^ physiological functions in aging models, and (ii) consequent changes in PrP^C^ biochemical properties. The latter may be triggered by the cellular aging process and may promote prion aggregate formation. To date, studies into PrP^C^ role in aging are contrasting, mostly due to different animal/cellular systems employed and the lack of consistency in defining aging in experimental models.

Here we provide a comprehensive overview of studies attempting to understand PrP^C^ function and fate in aging and relate them to the neurodegenerative processes.

## Aging

In mammals, aging is defined as the accumulation of changes in an individual over time and represents a multifaceted process of social, psychological, and physical alterations (Bowen and Atwood, 2004). Normal aging is associated with significant cognitive declines, such as decreased speed of information processing, working memory capacity, and long-term memory function (Hedden and Gabrieli, 2004). However, changes that occur in aging brains are less linked to chronological time than it was thought in the past. For instance, individual brains may grow old developing less alterations than others (Esiri, 2007).

The study of age-related changes in the human brain is challenging for two main reasons: (i) increasing improvement of nutrition during the last century limits our ability to directly compare human brain samples between generations; and (ii) most elderly brains show some pathological hallmarks, thus complicating the distinction between “normal aging” and “disease” (Esiri, 2007). In the past, it was thought that the main cause of age-related cognitive decline was massive neuron loss (Brody, 1955) and deterioration of dendritic branches (Scheibel et al., 1976; Scheibel, 1979). Now we know that alterations occurring in normal aging are more subtle and selective. In fact, it seems that most age-linked behavioral impairments are due to region-specific changes in dendritic morphology, cellular connectivity, calcium dysregulation, gene expression or other factors that affect plasticity and alter neuron network dynamics (Burke and Barnes, 2006). A crucial factor for brain aging is the enormous requirement that neurons have for oxidative metabolism required by energy consumption. Neurons need extraordinary amounts of energy because of: (i) the size and the ensuing energy-consumption system of transport for molecules and organelles; and (ii) the electrical activity for impulse transmission, implying ion gradient maintenance. The high degree of oxidative metabolism generates high amounts of ROS that damage proteins, nucleic acids and lipids, thus interfering with normal cell metabolism and resulting, for instance, in altered gene expression and abnormal protein generation (Esiri, 2007).

Mitochondrial efficiency decline, paralleled by oxidative stress, which occurs with aging, is connected to alterations in calcium homeostasis, in particular to higher calcium content in neuron cytosol after excitatory stimulation. Indeed, glutamate activation of N-methyl-D-aspartate (NMDA) receptors produces a transient elevation in intracellular calcium concentration, which is increased in normal aging. Disruption in calcium homeostasis predisposes aging neurons to more extended damage after stress and eventually leads to cell death by apoptosis (Cowan et al., 2001; Thibault et al., 2001; Esiri, 2007).

In this scenario, PrP^C^ may possess different functions: (i) neuron protection from oxidative stress through antioxidant activity, by sensing copper and/or free radical stimuli (Vassallo and Herms, 2003); (ii) modulation of calcium entry through NMDA receptor pore by inhibition of the channel activity (Cowan et al., 2001; Lo et al., 2007); (iii) anti-apoptotic effect on Bax-mediated cell death (Bounhar et al., 2006; Lo et al., 2007).

### Which mouse age should be considered as an aging model?

For many reasons, researchers often use terms as *aged* or *old mice* that may not be proper by definition. A wild-type mouse goes through the following life phases (http://research.jax.org/faculty/harrison/ger1vLifespan1.html) (Flurkey et al., 2007):

- Mature adult [3–6 months old (mo) mouse]: this period is the reference for any age change; these mice are fully developed, but not senescent; after 6 months of age mice can show some age-related changes, for instance females are retired from breeding because litter size begins to diminish.
- Middle age (10–15 mo mouse): senescent changes can be detected in some, but not all, biomarkers of aging; these mice are generally used to determine if an age-related change is progressive or occurs only in old age.
- Old (18–24 mo mouse, or older): senescent changes can be detected in almost all biomarkers in all animals.

Taking into account these categories, few studies claiming the use of old mice indeed employ a properly aged model. For the purposes of this manuscript, this point has to be carefully taken into account, especially in respect of studies on PrP^C^ biochemical property changes in aging. On the other hand, studies on PrP^C^ knockout mice (also noted as *Prnp*^0/0^), when not performed on old animals, as defined above, can nevertheless reveal progressive age-related changes that start earlier because of PrP^C^ absence. To let the reader critically consider reported results and conclusions, we will specify for each reviewed study the age of the animals used.

## PrP^C^ role in aging

As previously mentioned, PrP^C^ is highly conserved and expressed among mammals, and it may play important physiological roles. However, numerous efforts aimed at identifying PrP^C^ function have harbored contrasting results. Most of the work has focused on the nervous system, which is the organ with the highest PrP^C^ expression and the site of prion disease pathology. Several results have linked PrP^C^ to many cellular processes, such as neuronal survival, neurite outgrowth, synapse formation/maintenance/functionality, and myelinated fiber formation/maintenance (Aguzzi et al., 2008). However, its expression in many other tissues indicates that PrP^C^ has either many different functions or a function compatible with diverse cellular types.

A large body of evidence suggests that PrP^C^ plays a role in essential metal homeostasis, resulting in protection from oxidative stress. An overlap between systems controlling essential metal homeostasis and oxygen radical metabolism has been extensively documented (Avery, 2001). For instance, many antioxidant enzymes need metal ions as cofactors. PrP^C^ has been associated with cellular systems that control redox balance and protect against oxidative stress (Brown and Sassoon, 2002). Indeed, PrP^C^-null models show: (i) increased neuronal sensitivity to oxidative stress (Brown et al., 1997; Rachidi et al., 2003); (ii) alterations of superoxide dismutase 1 (SOD1) activity due to impairments in copper incorporation (Brown and Besinger, 1998; Kralovicova et al., 2009); (iii) higher levels of lipids and protein oxidation (Wong et al., 2001). Moreover, PrP^C^ expression is increased by heat shock, hypoxia, ischemia, hypoglycemia, stroke, and knockdown of any SOD protein while shutting down PrP^C^ increases extracellular-SOD expression and SOD2 activity (Brown and Besinger, 1998; Shyu et al., 2000, 2004, 2005; McLennan et al., 2004; Mitsios et al., 2007; Kralovicova et al., 2009). Furthermore, *in vitro* PrP^C^ possesses SOD-like activity with a dismutation constant rate similar to SOD2 (Brown et al., 1999, 2001; Cui et al., 2003; Treiber et al., 2007). A possible link between SOD and PrP^C^ may be copper. Indeed, SOD activity depends on copper incorporation and probably involves Cu^2+^ reduction, characteristics shared with PrP^C^ (Brown et al., 2001). It should also be noted that PrP^C^ is cleaved at the end of the OR through the action of ROS, a process termed β-cleavage. β-cleavage is considered an early and critical event in the mechanism through which PrP^C^ protects cells against oxidative stress. If a PrP construct lacks the OR, the protein will fail to undergo ROS-mediated β-cleavage, as occurs with two mutant forms associated with prion diseases (Watt et al., 2005).

### PrP^C^ role in behavior and learning as a function of age

Two main observations suggest that PrP^C^ plays a role in behavior and learning mechanisms in aging: (i) PrP^C^ is predominantly expressed in neurons, reaching the highest level in the hippocampus (DeArmond et al., 1987; Benvegnu et al., 2010); (ii) PrP^C^ brain expression is increased in aging (Williams et al., 2004). However, studies carried out on PrP^C^ knockout mice have sometimes produced contrasting results, likely due to differences in lines and age.

The behavioral characterization of the first PrP^C^ knockout (ZurichI) mouse generation was performed in 7 mo mice (Bueler et al., 1992). At this age these mice cannot be considered *aged*. Nevertheless, the latter study represents a good starting point toward analyzing alterations triggered by PrP^C^ ablation. In the swim test and in the Y-maze, PrP^C^ knockout mice did not reveal learning impairments, although there were large individual differences within test and control groups. In the two-way avoidance test, in which animals have to avoid an electrical shock announced by a light by running to the opposite chamber of the box, thus involving also motorial ability, PrP^C^ knockout mice showed a slightly lower performance, but the great variability in each group hid a possible significant difference. As Büeler and colleagues state, these results should be carefully considered because of the dissimilar loci surrounding *Prnp*^0/0^ and wild-type (*Prnp*^+/+^) alleles, due to different genetic background.

However, a follow-up study using the same mouse line showed that *Prnp*^0/0^ mouse aging-related behavior alterations could not be ascribed to surrounding loci differences, but to PrP^C^ ablation itself (Coitinho et al., 2003). Differently from Büeler and colleagues data, here statistically significant results were obtained by applying other behavioral tests and by increasing the animal age: 9 and 3 mo *Prnp*^0/0^ and *Prnp*^+/+^ mice were compared in regards to fear-motivated learning, locomotor activity, exploratory behavior and anxiety. Nine mo PrP^C^ knockout mice showed impairments in short- and long-term memory and exploratory activity. With exception of the exploratory activity, 9 mo *Prnp*^0/0^ mouse performances were recapitulated in 9 mo rats injected in the hippocampus with anti-PrP^C^ antibody, thus ruling out the involvement of the surrounding loci in memory alterations in the aging PrP^C^ knockout line.

Another study concerning aging-related PrP^C^ role in behavior control was performed on 3 and 11 mo mice from three lines (wild-type, *Prnp*^0/0^ and PrP^C^ overexpressing Tga20 on a *Prnp*^0/0^ genetic background) (Rial et al., 2009). Collectively the data showed that PrP^C^ overexpressing Tga20 mice were less susceptible to aging-caused alterations in locomotion, anxiety like responses and short-term social recognition memory in comparison with *Prnp*^+/+^ and *Prnp*^0/0^ mice. On the contrary, PrP^C^ knockout mice resulted more susceptible to age-related decline in comparison with wild-type mice. Biochemical analyses revealed that Tga20 mice have lower neuron caspase3 activation and serum acetylcholinesterase levels, and higher synaptic density. In light of these cognition-enhancing properties, Rial and colleagues suggest in particular the interaction of PrP^C^ with the stress-inducible protein 1 as a target for pharmaceutical intervention to attenuate age-related cognitive impairments.

More recent results confirmed age-dependent behavioral abnormalities in ZurichI PrP^C^ knockout mice (Schmitz et al., 2014). The following alterations were reported: (i) poor native nest building behavior in young (3 mo) and old (9 and 20 mo) PrP^C^ knockout mice; (ii) higher latency in exploring a new environment only in young but not in old PrP^C^ knockout mice; (iii) more pronounced drop in anxiety during aging in PrP^C^ knockout mice; (iv) decline in associative learning in old PrP^C^ knockout mice compared to age-matched wild-type. Schmitz and colleagues wanted to relate the observed behavioral abnormalities to neuron structural alterations in PrP^C^ knockout mice. They found PrP^C^-dependent alterations in cytoskeletal proteins that are responsible for morphology, structure, and stability of neurons, thus related to learning processes. Indeed, PrP^C^ was previously reported to functionally interact with cytoskeletal proteins (Dong et al., 2008; Malaga-Trillo et al., 2009).

Telling's group performed a work on a slightly different line, the ZurichI crossed with wild-type FVB animals (Nazor et al., 2007). On the rotarod, *Prnp*^0/0^ on FVB background mice showed an age-dependent motor behavior deficit in comparison to wild-type, suggesting a function for PrP^C^ in maintaining sensorimotor coordination. This difference between wild-type and PrP^C^ knockout mice was detected starting from 3 to 8 mo. Moreover, these mice at 6 mo revealed vacuolation in different brain regions. Although vacuolation has been rarely reported in PrP^C^ knockout mice, Telling and coworkers parallel their findings, concerning both spongiosis and motor impairments, with results from a transgenic murine model expressing the PrP mutation linked to Gerstmann–Sträussler–Scheinker syndrome (GSS), thus suggesting the loss of PrP^C^ function as disease mechanism.

Another study was performed by Massimino and colleagues using three groups of congenic mice: wild-type FVB, PrP^C^ knockout on FVB background and Tg46 mice in which PrP^C^ expression was rescued over a PrP^C^ knockout background (Massimino et al., 2013). While young (3 mo) PrP^C^ knockout mice did not show any alteration, aged (15–18 mo) animals revealed perturbed behavioral pattern, in particular difficulties in adapting to new situations and in locomotor activity, likely due to a depressive syndrome. The authors suggest that PrP^C^ absence affects emotional reactivity. The authors did not carry out histological analysis of old *Prnp*^0/0^ mice, therefore, it is not known whether the PrP^C^ knockout colony employed showed the vacuolation phenotype observed by Telling's group studying the same mouse line. To our knowledge, the result obtained by Telling's and coworkers is the only one showing vacuolation in *Prnp*^0/0^ mice. For instance, Aguzzi and colleagues did not detect any morphological anomaly in 14 mo ZurichI *Prnp*^0/0^ mice (Bremer et al., 2010).

Taken together, all these studies demonstrate that PrP^C^ absence affects learning, cognitive and behavioral skills in aged mice. A possible cause of these alterations is the lack of PrP^C^-mediated neuroprotection and reduction of oxidative stress, particularly important to preserve neuron functions in the aging brain milieu.

### PrP^C^ role in myelin maintenance

Alterations occurring during both normal aging and neurodegenerative pathologies largely involve maintenance and regeneration of myelin structure (Verdu et al., 2000; Peters, 2002; Bartzokis, 2011). Metal ions, in particular copper and iron, are necessary for both myelin formation and maintenance (Skripuletz et al., 2008; Benetti et al., 2010). Therefore, PrP^C^ may influence myelin by maintaining copper and iron homeostasis, pathways in which PrP^C^ has been involved (Pauly and Harris, 1998; Brown and Harris, 2003; Rachidi et al., 2003; Miura et al., 2005; Singh et al., 2009). By studying 14 mo (middle aged) mice, Aguzzi's group reported that PrP^C^ knockout mouse sciatic nerve contains more *digestion chambers*, i.e., macrophages ingesting myelin debris of degenerating nerve fibers, compared to wild-type mice. Moreover, they showed that PrP^C^ expression on neuronal cells, but not on Schwann's cells, is fundamental to preserve myelin fibers and prevent chronic demyelinating polyneuropathy (CDP) in the peripheral nervous system. This finding suggests that neuronal PrP^C^ *in trans* expression is required for a directional communication from axons to Schwann cells (Bremer et al., 2010).

As suggested in a study from our group (Benvegnu et al., 2011), PrP^C^ may exert a direct role in myelin sheath formation and structural preservation through its modulation of β-secretase 1 (BACE1) (Parkin et al., 2007). Results obtained in sciatic nerves from 15 mo mice highlight that PrP^C^ influences cleavage and processing of neuregulins. Neuregulins are a class of proteins crucial for myelin maintenance in the peripheral nervous system, and their cleavage by BACE1 is necessary for signaling functions. Hence, PrP^C^ positively regulates neuregulin processing, thus affecting their function in myelin homeostasis, maybe by modulating BACE1 activity. Interestingly, in the previously mentioned work, Aguzzi's group showed that CDP is prevented by PrP^C^ variants that are permissive for proteolytic amino-proximal cleavage, but not by variants that do not undergo cleavage. Indeed, all transgenic mice showing CDP lacked C1, i.e., the fragment generated by α-cleavage, while all transgenic mice in which the CDP was rescued produced high C1 levels. Hence, the cleavage of PrP^C^ seems to be functional for its myelinotrophic role (Bremer et al., 2010).

## Aging-related modification of PrP^C^ biochemical properties

The most common occurrence of human prion diseases (about 80%) is sporadic. This means that a trigger of prion pathologies may be neither a genetic mutation nor an infectious seed, but most likely an unknown alteration that provokes the switch from PrP^C^ to PrP^Sc^ (Prusiner, 2001). The aging process itself may modify PrP^C^ biochemical properties, making the protein more prone to convert into PrP^Sc^. For instance, loss of glycosylation in cell models favors PrP^C^ acquisition of PrP^Sc^-like features (Lehmann and Harris, 1997). Indeed, glycans regulate PrP^C^ folding, intracellular trafficking, localization and function on the neuronal surface, thus their pattern modifications can be likely linked to the disease development. PrP^C^ biochemical properties that may be altered by cellular changes occurring during the aging process are: expression levels, post-translational modifications, such as glycosylation and phosphorylation, as well as localization in cellular domains, such as inside/outside membrane lipid rafts.

As mentioned in a previous paragraph, analysis of both parenchyma and microvessels revealed higher levels of both glycosylated and unglycosylated PrP^C^ in C57Bl/6J old (18 and 24 mo) mice (Williams et al., 2004). Concerning PrP^C^ diverse glycosylation isoforms, the three main bands appearing in one-dimensional immunoblots are commonly considered corresponding to di-glycosylated (~35 KDa), mono-glycosylated (~32 KDa) and un-glycosylated (~28 KDa) (Collinge, 2001; Pan et al., 2002). However, by applying a panel of monoclonal antibodies, the presence of additional lower molecular weight bands representing N-terminally truncated PrP^C^ isoforms has been shown (Pan et al., 2002). Moreover, two-dimensional immunoblot analysis revealed in human brain more than 50 PrP^C^ species which derive from several glycosylation and cleavage combinations. Interestingly, accumulation of aberrant full-length PrP^C^ bound to immature N-linked glycans is indeed a common feature of prion disease (Pan et al., 2005a,b,c). Spurred by these findings, Goh and coworkers investigated N-linked glycans on PrP^C^ during normal aging in mouse, providing the first glycan profile of full-length and truncated PrP^C^ isoforms (Goh et al., 2007). The oldest mice they used were 15 mo. First, they found that different mouse lines (i.e., CD-1 and FVB) have different PrP^C^ metabolism resulting in isoform heterogeneity. In general, they showed that the truncated form of PrP^C^ undergoes a simplification in its glycosylation process during aging, while the amount of complex oligosaccharides on the full-length PrP^C^ increases. This finding contrasts with what was observed in aging compared to young cattle (Yoshioka et al., 2010), but resembles what was previously detected in human brains affected by prion disorders (Pan et al., 2005b). It has been shown that PrP^C^ glycosylation state may modulate affinity for copper binding (Moudjou et al., 2007). In particular, the non-glycosylated species showed stronger binding to divalent cations (copper and cobalt) *in vitro*. Therefore, decreasing PrP^C^ glycosylation levels during aging may increase copper binding capacity, thus improving PrP^C^ antioxidant function. Interestingly, galactose, which has been detected in this study on full-length PrP^C^ in aging, is also highly present on PrP^Sc^ (Safar et al., 1990). Moreover, sialic acid content increases with aging on the truncated PrP^C^ but remains unchanged on the full-length PrP^C^. As the authors observe, the presence of anionic residues may modulate PrP^Sc^ mobility, thus facilitating its propagation in prion disorders.

As previously mentioned, aging can modify PrP^C^ subcellular localization. Indeed, our group reported that PrP^C^ moves from detergent soluble membrane fractions to lipid rafts in aged (20–21 mo) mouse hippocampus (Agostini et al., 2013). No differences in the total PrP^C^ expression amount were detected, in contrast with what had been previously reported by another group that used the same mouse line and a very similar age but lacked repeated samples, normalization and statistical analysis (Williams et al., 2004). Changes in the cholesterol/sphingolipid ratio has been reported in normal brain aging, and in degenerative disorders, such as AD (Martin et al., 2010). As cholesterol and sphingolipids are the two main components of lipid rafts, their relative amount influences many cellular pathways, including protein localization (Martin et al., 2008; Trovo et al., 2011). PrP^C^ is a GPI-anchored protein, thus bound to lipid rafts and likely affected by aging-related alterations in membrane composition. PrP^C^ shift toward lipid rafts triggered by changes in lipid composition was confirmed using model systems of lipid manipulation. Moreover, decreasing sphingolipids, thus mimicking a juvenile condition, reduced the formation of PrP^Sc^ in a cell line model. This suggests that age-related changes influence PrP^C^ localization and ensuing propensity to PrP^Sc^ conversion (Agostini et al., 2013).

## PrP^C^ and AD

The most prevalent form of dementia is AD (Burns and Iliffe, 2009). Various factors are conducive to the risk of late-onset AD, in particular old age, genetic factors, family history, a history of head trauma, midlife hypertension, obesity, diabetes, and hypercholesterolemia (Bendlin et al., 2010). AD is characterized by the misprocessing of two proteins: intraneuronal tau and extracellular β-amyloid peptide (Aβ) (Querfurth and LaFerla, 2010). Aβ is generated by the aberrant proteolytic processing of the amyloid precursor protein by BACE1. Several studies have been performed to understand if a toxic interaction between Aβ and PrP exists, but the use of different *in vitro* or transgenic models has yielded contrasting results (Schwarze-Eicker et al., 2005; Lauren et al., 2009; Balducci et al., 2010; Calella et al., 2010; Chung et al., 2010; Kessels et al., 2010; Morales et al., 2010; Ordonez-Gutierrez et al., 2013). Thus, in this manuscript, we will consider only what has been found by analyzing AD patient brains. Whitehouse and colleagues found that PrP^C^ levels are decreased in AD patient hippocampus, normal aging hippocampus, and temporal lobe, but not in AD patient temporal lobe (Whitehouse et al., 2010). With exception of AD patient temporal lobe data, these results are in contrast with what was reported by Saijo et al. (2011), but correlate with the PrP^C^ decrease detected in the cerebrospinal fluid of patients affected by different neurological disorders including AD (Meyne et al., 2009). Since PrP^C^ inhibits BACE1 (Parkin et al., 2007), PrP^C^ level reduction may elevate Aβ production. Therefore, Whitehouse and colleagues suggest that PrP^C^ decrease is not a secondary consequence, but a primary cause of AD and, by occurring also in normal aging, increases the incidence of AD in old individuals. Interestingly, individuals with mutations in the PrP encoding gene generating a truncated form of the protein developed clinical AD symptoms at relatively young age (Kitamoto et al., 1993). In aging, BACE1 activity strongly increases, thus enhancing Aβ production and the possibility of deposit formation. The reduction of PrP^C^-mediated BACE1 inhibition due to the decrease in PrP^C^ levels may be a cause of the enzyme activity elevation (Whitehouse et al., 2010). Additionally, since PrP^C^ is an antioxidant protein (Vassallo and Herms, 2003; Aguzzi et al., 2008), its downregulation likely increases neuron susceptibility to ROS, which rises in normal aging and in AD (Halliwell, 2006; Zhu et al., 2007), hence contributing to disease progression. However, a decrease in PrP^C^ levels in AD does not exclude that the residual protein mediates some Aβ toxic effects. Besides, it has been recently reported that Aβ and PrP^C^ do interact specifically in AD patient brains (Dohler et al., 2014). This result was obtained by means of a PrP^C^-Aβ binding assay and size exclusion chromatography, and no binding was detected in non-demented age-matched controls.

Taken together, these results indicate that PrP^C^ may be involved in AD. Therefore, its fate in aging is likely related to the molecular mechanisms that induce neurodegeneration.

## Conclusions

As presented in this review, relatively few studies have been carried out on the role of aging in the expression and regulation of PrP^C^. Nevertheless, these experiments suggest that there may be a correlation between the physiology of the cells in which PrP^C^ is present and its localization and processing during aging. Particularly in the CNS, where it is abundantly expressed, PrP^C^ plays a prominent role as the precursor of PrP^Sc^ and thereby dictates the amount and extension of the conversion and accumulation process of prions. In aging, the physiology and the cellular localization of the protein may change concomitantly to different biochemical milieus in the cell membrane. Indeed, either membrane composition, in particular lipid raft composition, or additional protein complexes proximity to PrP^C^, may influence its physiological functions. As indicated in the final paragraph, these changes may have a general relevance for more common causes of dementia such as AD. More work is necessary to define the precise role of PrP^C^ in the progression of AD and perhaps in other neurodegenerative diseases.

### Conflict of interest statement

The authors declare that the research was conducted in the absence of any commercial or financial relationships that could be construed as a potential conflict of interest.
